# Comparative study of corticosteroid monotherapy, and TNF inhibitors with or without corticosteroid in patients with refractory entero-Behcet’s disease

**DOI:** 10.1186/s13075-019-1933-8

**Published:** 2019-06-22

**Authors:** Ippei Miyagawa, Kazuhisa Nakano, Shigeru Iwata, Shingo Nakayamada, Kazuyoshi Saito, Kentaro Hanami, Shunsuke Fukuyo, Satoshi Kubo, Akio Kawabe, Yusuke Miyazaki, Yoshiya Tanaka

**Affiliations:** The First Department of Internal Medicine, School of Medicine, University of Occupational and Environmental Health, 1-1 Iseigaoka, Yahata-nishi, Kitakyushu, 807-8555 Japan

**Keywords:** Behcet’s disease, Entero-Behcet’s disease, TNF inhibitor, Corticosteroid, Disease activity index

## Abstract

**Background:**

Tumor necrosis factor (TNF) inhibitors (TNF-i) are effective in the treatment of entero-Behcet’s disease (BD). However, there is no objective tool for assessment of disease activity in entero-BD; therefore, it is not easy to evaluate treatment effectiveness in the clinical setting. In addition, because corticosteroid (CS) is considered for standard therapy, the effectiveness of TNF-i without CS has not been well examined. In this retrospective study, the effectiveness of CS without TNF-i and the effectiveness of TNF-i with or without CS therapy were investigated and compared.

**Methods:**

This study included 71 patients with entero-BD who were followed up for 1 year (CS without TNF-i group: *n* = 22; TNF-i group: *n* = 49 [with CS: *n* = 20, without CS: *n* = 29]). All patients had active ulcerative lesions. The primary endpoint was the ulcer cure rate evaluated by lower gastrointestinal endoscopy. Secondary endpoints were ulcer improvement rate, disease activity improvement based on the quantitative disease activity index for intestinal Behcet’s disease (DAIBD), and CS-sparing effect.

**Results:**

Ulcer cure rates were 13.6% in the CS without TNF-i group, 60.0% in the TNF-i with CS group, and 44.8% in the TNF-i without CS group. Ulcer improvement rates were 27.2% in the CS without TNF-i group, 60.0% in the TNF-i with CS group, and 51.7% in the TNF-i without CS group. The multivariate analysis revealed that TNF-i was an independent predictive factor for cure of the ulcerative lesions. The DAIBD and concomitant CS dose were significantly decreased in both the CS without TNF-i group (DAIBD 85.2 → 40.5, CS 32.3 → 18.7 mg/day) and the TNF-i group (DAIBD 64.7 → 21.1. CS 18.7 → 3.88 mg/day). The ulcer cure and improvement rates were significantly higher in the TNF-i group. In addition, the proportion of concomitant CS dose less than 7.5 mg was significantly higher in the TNF-i group (CS without TNF-i group 18.2% vs. TNF-i group 85%, *P* < 0.01). There were no statistically significant differences between the TNF-i with CS group and the TNF-i without CS group in any of the endpoints.

**Conclusions:**

This study demonstrated that compared to CS alone, TNF-i improve disease activity and possess a higher ulcer healing effect and CS tapering effect with or without concomitant CS.

## Background

Behcet’s disease (BD) is a relapsing, systemic inflammatory disease that affects the skin, mucosa, eyes, genitourinary organs, joints, blood vessels, central nervous system, and gastrointestinal system [1]. Entero-Behcet’s disease (entero-BD) is often associated with bleeding and perforation, which is refractory to therapy. Many patients with entero-BD are resistant to conventional treatment with corticosteroid (CS) and immunosuppressive drugs, and some require surgery. We and other groups have recently shown that TNF inhibitors (TNF-i) are effective in the treatment of entero-BD [2–8]. However, there is no objective tool for assessment of disease activity in entero-BD; therefore, it is not easy to evaluate disease activity and treatment efficacy in the clinical setting. In addition, because CS is considered for standard therapy in patients with severe symptoms and with deep ulcerative lesions [9, 10], the effectiveness of TNF-i without CS has not been well examined [11]. In the present study, we quantitatively investigated the effectiveness of 1-year treatment with TNF-i in 49 patients with entero-BD refractory to conventional therapy and compared the results to those achieved with CS without TNF-i, using the disease activity index for intestinal Behcet’s disease (DAIBD) developed in 2011 [12, 13]. We also investigated effectiveness of TNF-i without CS and compared to that of TNF-i with CS.

## Patients and methods

### Patients

The present study included 71 patients diagnosed with entero-BD according to the revised diagnostic criteria proposed by the Behcet’s Disease Research Committee of Japan (2003) and the criteria for diagnosis of BD recommended by the International Study Group [14, 15]. All 71 patients had active ulcerative lesions. Patients with diseases similar to BD, such as intestinal tuberculosis, Crohn’s disease, ulcerative colitis, infectious colitis, or simple ulcers, were excluded. Patients who were newly introduced the remission induction therapy mainly with CS but without TNF-i for active ulcerative lesions at our hospital were defined as CS without TNF-i group, regardless of past and recent treatment and dose of concomitant CS. Likewise, patients newly introduced the remission induction therapy with TNF-i for active ulcerative lesions at our hospital were defined as the TNF-i group, regardless of past and recent treatment and use and dose of concomitant CS. Choices regarding type of TNF-i and use and dose of CS were made at the physicians’ discretion. Remission induction therapy was defined as the treatment for newly emerging active ulcerative lesions. This study formed part of a research project investigating BD supported by the Health Labour Science Research Grant for research on rare and intractable diseases. The Human Ethics Review Committee of our university reviewed and approved this study. All participants provided informed consent prior to inclusion in the study. Details that might disclose the identity of study subjects were omitted.

### Clinical measurement

In the present study, we investigated the effectiveness of 1-year treatment with TNF-i and compared the results to those achieved with CS without TNF-i. We also investigated effectiveness of TNF-i without CS and compared to that of TNF-i with CS.

**Fig. 1:**
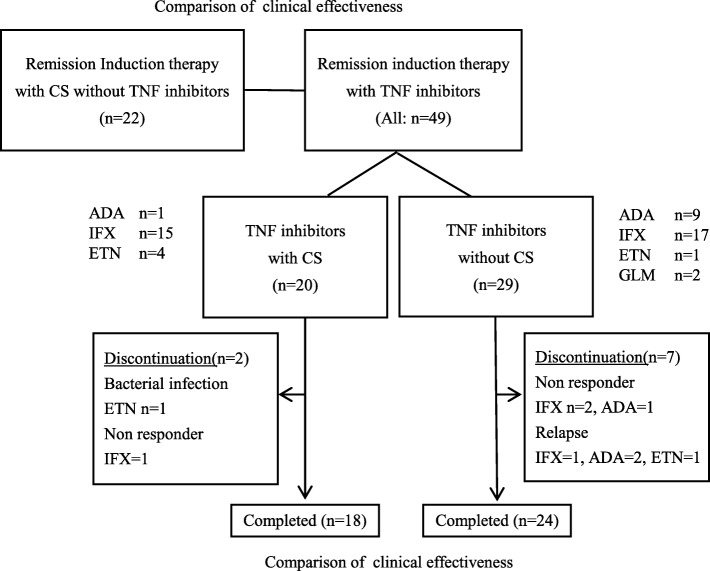
Study design

Remission induction therapy mainly with CS but without TNF-i (22 patients) and remission induction therapy with TNF-i (49 patients; 20 TNF-i with CS, 29 TNF-i without CS) were newly introduced at our hospital. Study patients were followed up for 1 year after the introduction of the treatments at our hospital and affiliated institutions, as shown in Fig. 1. The primary endpoint was the ulcer cure rate, as evaluated by lower gastrointestinal endoscopy 1 year after the introduction of remission induction therapy with either TNF-i or CS without TNF-i. “Cure” was defined as disappearance and scarring of ulcerative lesions. “Improvement” was defined as cure, scarring, and reduction of ulcerative lesions. In the case of patients who were unable to undergo lower gastrointestinal endoscopy owing to abdominal pain or for other reasons, the status was classified as “Exacerbation.” In the TNF-i without CS group, in patients for whom concomitant CS was newly introduced, the status was classified as “Exacerbation.” Likewise, in the CS without TNF-i group, in patients for whom concomitant CS was increased, the status was classified as “Exacerbation.” The secondary endpoints were the ulcer improvement rate, ulcer exacerbation rate, and continuation rate of TNF-i. Regarding the continuation rate of the TNF-i without CS group, in patients for whom CS was newly introduced, the status was classified as “Discontinuation.” We also analyzed disease activity improvement based on the DAIBD and the CS-sparing effect after 1 year of treatment.

The DAIBD (Disease Activity Index for intestinal Behcet’s disease) is a quantitative disease activity index proposed by Cheon et al. in 2011 [14, 15]. Prior to the development of the DAIBD, there had been no useful and objective tool to assess the disease activity of entero-BD. The DAIBD (total score, 0–285) consists of eight items: (i) general well-being for 1 week, (ii) fever, (iii) extra-intestinal manifestations, (iv) abdominal pain in 1 week, (v) abdominal mass, (vi) abdominal tenderness, (vii) intestinal complications, and (viii) number of liquid stools in 1 week.

### Statistical analysis

Data are expressed as mean ± standard deviation (SD). Differences between the groups and between baseline data and data measured at 1 year were compared using Fisher’s exact test, the Mann–Whitney *U* test, and the Wilcoxon signed-rank test. The patients were classified into groups with cure of intestinal ulcerative lesions or not, or with achieving CS less than 7.5 mg/day group or not. In addition, univariate and multivariate logistic regression analyses were performed on these groups. In the multivariate analysis, the proportion of cure of intestinal ulcerative lesions or achieving CS less than 7.5 mg/day at year 1 were used as a dependent variable. All reported *P* values were two-sided. The level of significance was set at *P* < 0.05.

All analyses were conducted using JMP version 10.0 (SAS Institute Inc., Cary, NC, USA). For statistical analysis, data from TNF-i discontinued cases were complemented using the last observation carried forward method.

## Results

### Patient background

Table 1 shows the patient background at baseline. All patients in the present study were diagnosed with entero-BD based on the existence of the typical ulcerative lesions on gastrointestinal endoscopy. In the group with CS without TNF-i (22 patients), the mean patient age was 34.1 years. The median dose of concomitant CS was 30 mg/day (prednisolone equivalent). The mean DAIBD was 85.2. In the group with TNF-i and CS group (20 patients), the mean patient age was 42.2 years. The median dose of concomitant corticosteroid was 10 mg/day (prednisolone equivalent). The mean DAIBD was 70.7. In contrast, in the group with TNF-i without CS (29 patients), the mean patient age was 40.7 years. Twenty-one patients had no history of TNF-i treatment (Bio naive). The mean DAIBD was 60.5. Although the concomitant CS dose and the DAIBD were statistically significantly higher in the group with CS without TNF-i than in the group with TNF-i, there were almost no statistically significant differences in baseline background factors between the TNF-i with CS group and the TNF-i without CS group.

**Table 1:** Baseline characteristics of 71 patients with entero-Behcet’s disease

	With TNF inhibitors (all: *n* = 49)			With CS without TNF inhibitors	
	With CS (*n* = 20)	Without CS (*n* = 29)	*P* value*	(*n* = 22)	*P* value**
Male, *n* (%)	5 (25)	7 (24.1)	0.6607	10 (45.5)	0.0773
Age	42.2 ± 13.4	40.7 ± 14.7	0.6989	34.1 ± 11.8	0.0349
Clinical manifestations at diagnosis, *n* (%)	Recurrent aphthae 20 (10),	Recurrent aphthae 29 (100),		Recurrent aphthae 22 (100),	
	Skin 20 (100), uveitis 3 (15), genital ulcer 14 (70), arthritis 10 (50), epididymitis 1 (5), digestive tract sores 20 (100), vascular lesion 5 (25), CNS lesion 3 (15)	Skin 26 (89.7), uveitis 3 (10.3), genital ulcer 19 (65.5), arthritis 21 (72.4), digestive tract sores 29 (100), vascular lesion 1 (3.4), CNS lesion 4 (13.8)		Skin 22 (100), uveitis 7 (31.8), genital ulcer 13 (59.1), arthritis 18 (81.8), digestive tract sores 22 (100), vascular lesion 2 (9.1), CNS lesion 3 (13.6)	
Type, *n* (%)	Complete 3 (15), incomplete 17 (85)	Complete 3 (10.3), incomplete 26 (89.7)	0.8244	Complete 2 (9.1), incomplete 20 (90.9)	0.6975
Vascular-BD, *n* (%)	5 (25)	None	0.0081*	2 (9.1)	0.9163
Neuro-BD, *n* (%)	2 (10)	8 (27.6)	0.1260	3 (13.6)	0.4951
HLA-B51 positive (%)	4 of 18 (22.2)	6 of 20 (30)	0.4323	3 of 10 (30)	0.8156
Disease duration BD (month)	83.8 ± 73.5	86.6 ± 67.0	0.6451	48.2 ± 30.0	0.0552
entero-BD (month)	55.8 ± 69.3	50.1 ± 56.6	0.6540	28.3 ± 21.2	0.5135
Site of ulceration, *n* (%) (overlapping)	Ileum 4 (20), ileocecum 13 (65), ascending colon 5 (25), transverse colon 2 (10), descending colon 4 (20), sigmoid colon 4 (20)	Ileum 6 (20.7), ileocecum 15 (51.7), ascending colon 8 (27.6), transverse colon 7 (24.1), descending colon 5 (17.2), sigmoid colon 7 (24.1), esophagus 1 (3.4)		Ileum 4 (18.2), ileocecum 17 (77.3), ascending colon 5 (22.7), transverse colon 4 (18.2), descending colon 2 (9.1), sigmoid colon 1 (4.5)	
Cases with multiple ulceration, *n* (%)	7 (35)	10 (34.5)	0.9072	7 (31.8)	0.8128
Treatment history, *n* (%)	High-dose CS 9 (45), low-dose CS 9 (45), col 10 (50), MTX 17 (85), SSZ/MS 8 (40), AZ 3 (15), IVCY 2 (10), IFX 3 (15), GLM 3 (15), ETN 1 (5)	High-dose CS 3 (10.3), low-dose CS 3 (10.3), col 19 (65.5), MTX 16 (55.2), SSZ/MS 11 (37.9), AZ 3 (10.3), CsA 1 (34.4), IFX 5 (17.2), GLM 1 (34.5), ETN 3 (10.3), ADA 3 (10.3)		High-dose CS 13 (59.1), col 14 (63.6), MTX 1 (45.5), SSZ/MS 11 (50), AZ 1 (45.5)	
Bio naïve, *n* (%)	16 (80)	21 (69.0)	0.7605	NA	
History of relapse, *n* (%)	13 (65)	16 (55.2)	0.3488	11 (50)	0.4076
History of perforation, *n* (%)	5 (25)	4 (13.8)	0.2654	1 (4.5)	0.1216
History of surgery, *n* (%)	5 (25)	1 (3.4)	0.0595	2 (9.1)	0.6975
Concomitant CS dose (mg/day)	18.7 ± 20.4 median 10, range 2–62.5	NA		32.3 ± 16.4 median 30, range 6–60	0.0069**
Concomitant drugs	MTX 17 (85), col 4 (20), MS/SSZ: 3 (15), AZ 1 (5)	MTX 23 (79.3), col 14 (48.3), MS/SSZ 4 (13.8), AZ 3 (10.3)		MS/SSZ 11 (50), col 14 (63.6), AZ 1 (4.5), MTX 1 (4.5)	
Introduced TNF-i	IFX 15 (75), ETN 4 (20), ADA 1 (5)	IFX 17 (58.6), ETN 1 (3.4), ADA 9 (31.0), GLM 2 (6.9)		NA	
DAIBD	70.7 ± 38.4, median 62.5, range 50–93	60.5 ± 32.2, median 60.0, range 37.5–80	0.3045	85.2 ± 29.6, median 87.5, range 35–135	0.0139**
General well-being, *n* (%)	Fair 10 (50), poor 3 (15), very poor 1 (5)	Fair 19 (65.5)		Fair 18 (81.8)	
Fever (≧ 38 °C), *n* (%)	6 (30)	3 (10.3)		11 (50)	
Extra-intestinal manifestation, *n* (%)	Oral 12 (60), genital ulcer 7 (35), eye 0, skin 9 (45), arthralgia 5 (25), vascular 5 (25), CNS 1 (5)	Oral 10 (34.5), genital ulcer 7 (24.1), eye 0, skin 7 (24.1), arthralgia 8 (27.6), vascular 0, CNS 7 (24.1)		Oral 15 (68.2), genital ulcer 5 (22.7), eye 0, skin 16 (72.7), arthralgia 6 (27.2), vascular 2 (9.1), CNS 1 (4.5)	
Abdominal pain, *n* (%)	Mild 8 (40), moderate 6 (30), severe 1 (5)	Mild 11 (37.9), moderate 4 (13.8)		Moderate 18 (81.8), severe 2 (9.1)	
Abdominal mass, *n* (%)	1 (5)	None		None	
Abdominal tenderness, *n* (%)	Mildly 7 (35), moderately or severely 10 (50)	Mildly 14 (48.3), moderately or severely 4 (13.8)		Mildly 3 (13.6), moderately or severely 17 (77.3)	
Intestinal complication, *n* (%)	Perforation 4 (20), abscess 1 (5), obstruction 3 (15)	Perforation 1 (3.4)		None	
No of liquid stool in 1 week, *n* (%)	1–7 times: 6 (30), 8–21 times: 3 (15), 22–35 times: 3 (15)	1–7 times: 7 (24.1), 8–21 times: 2 (6.9), 22–35 times: 3 (10.3)		1–7 times: 2 (9.1), 8–21 times: 7 (31.8), ≧ 36 times: 4 (18.2)	

Data are shown by means ± SD or *n* (%). *P* value *< 0.05: TNF inhibitors with CS group (*n* = 20) vs. TNF inhibitors without CS group (*n* = 29), *P* value **< 0.05: TNF inhibitors group (all; *n* = 49) vs. CS without TNF inhibitors group (*n* = 22). *BD* Behcet’s disease, *CS* corticosteroid (prednisolone or equivalent), *HLA* human leukocyte antigen, *IVCY* cyclophosphamide pulse therapy i.v, *MS* mesalazine, *SSZ* sulfasalazine, *MTX* methotrexate, *AZ* azathioprine, *CsA* cyclosporine, *col* colchicine, *IFX* infliximab, *ADA* adalimumab, *GLM* golimumab, *ETN* etanercept, *DAIBD* disease activity index for intestinal Behcet’s disease, *NA* not applicable

### Treatment effectiveness as evaluated by lower gastrointestinal endoscopy

**Fig. 2:**
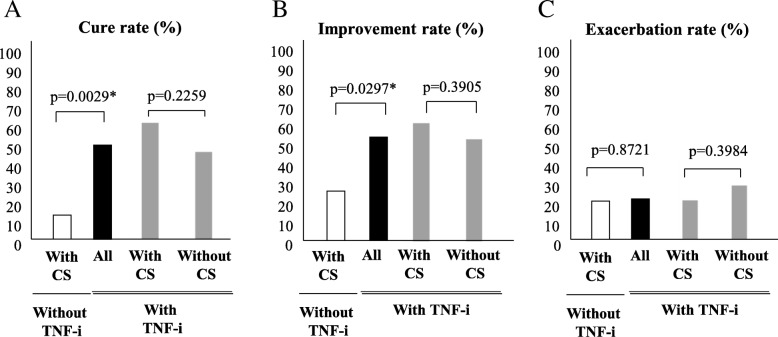
Treatment effectiveness as evaluated by lower gastrointestinal endoscopy. **a** Ulcer cure rate, **b** improvement rate, and **c** exacerbation rate as evaluated by lower gastrointestinal endoscopy in the CS without TNF-i group (*n* = 22), TNF-i group (all: *n* = 49), TNF-i with CS group (*n* = 20), and TNF-i without CS group (*n* = 29) (*P < 0.05, by Fisher’s exact test) (last observation carried forward). Abbreviations: CS, corticosteroid; TNF-i, TNF inhibitors; DAIBD, disease activity index for intestinal Behcet’s disease

The ulcer cure rate at 1 year was 13.6% (3 of 22 patients) in the group with CS without TNF-i. In the same group, the ulcer improvement rate was 27.3% (6 of 22 patients). In contrast, the ulcer cure rates at 1 year were 60.0% (12 of 20 patients) in the group with TNF-i and CS, and 44.8% (13 of 29 patients) in the group with TNF-i without CS. The ulcer improvement rates were 60.0% (12 of 20 patients) in the group with TNF-i and CS and 51.7% (15 of 29 patients) in the group with TNF-i without CS. Although the ulcer cure and improvement rates were significantly lower in the group with CS without TNF-i than in the group with TNF-i, there were no significant differences in these outcomes between the TNF-i with CS group and the TNF-i without CS group (Fig. 2a, b). The ulcer exacerbation rates were 20.0% (4 of 20 patients) in the group with TNF-i and CS and 29.8% (8 of 29 patients) in the group with TNF-i without CS (*P* = 0.3984) (Fig. 2c). During the observation period, three patients were newly started on CS in the group with TNF-i without CS, and the status in these patients was classified as “Exacerbation” and “Discontinuation.” The data of these patients were complemented using the last observation carried forward method. In the present study, the cure of the intestinal ulcerative lesions was observed in 28 patients. When baseline characteristics were compared between patients with and without cure of the intestinal ulcerative lesions, as shown in Table 2, TNF-i was significantly more frequently used in patients with cure of the intestinal ulcerative lesions. In addition, univariate and multivariate logistic regression analyses were performed to identify the predictive factors for the cure of ulceration. The univariate logistic analysis identified colchicine, MTX, and TNF-i use and history of perforation with a *P* value of < 0.1. Subsequently, a multivariate logistic analysis was performed with these factors as dependent variables and identified the TNF-i use as an independent predictive factor (*P* = 0.0416, odds ratio 6.17, 95% confidence interval 1.01–37.8) (Table 3).

**Table 2:** Comparison of baseline characteristics between cure of the ulcerative lesions group and not

Variables	Cure of the ulcerative lesions (*n* = 28)	Not (*n* = 43)	*P value
Male, *n* (%)	10 (35.7%)	12 (27.9%)	0.6011
Age (years)	40.2 ± 12.3	38.3 ± 14.6	0.2659
Disease duration (entero-BD, months)	49.6 ± 64.1	41.9 ± 45.6	0.8923
Multiple ulceration, *n* (%)	7/28 (25.0%)	17/43 (39.5%)	0.3048
History of relapse, *n* (%)	16/28 (57.1%)	24/43 (55.8%)	1.0000
History of perforation, *n* (%)	7/28 (25%)	3/43 (6.98%)	0.0425*
History of surgery, *n* (%)	5/28 (17.9%)	3/43 (6.98%)	0.2495
Concomitant CS use, *n* (%)	15/28 (53.6%)	27/43 (62.8%)	0.4686
Concomitant CS dose (mg/day)	14.5 ± 20.5	15.8 ± 19.2	0.5661
Concomitant colchicine use, *n* (%)	7/28 (25%)	23/43 (53.5%)	0.0265*
Concomitant drug use, *n* (%) (MTX, MS/SSZ, AZ)	26/28 (92.9%)	34/43 (79.1%)	0.1812
Concomitant MTX use, *n* (%)	21/28 (75%)	20/43 (46.5%)	0.0265*
TNF-i use, *n* (%)	25/28 (89.3%)	24/43 (55.8%)	0.0036*
DAIBD	67.3 ± 31.0	73.5 ± 36.7	0.6332

Plus-minus values are means ± standard deviation. *P < 0.05 by Fisher’s exact test or Mann–Whitney *U* test. *BD* Behcet’s disease, *CS* corticosteroid, *col* colchicine, *MS* mesalazine, *SSZ* sulfasalazine, *MTX* methotrexate, *AZ* azathioprine, *TNF-i* TNF inhibitors, *DAIBD* disease activity index for intestinal Behcet’s disease

**Table 3:** Baseline predictive factors for cure of ulcerative lesions by multivariate analysis

Variables	Univariate logistic regression			Multiple logistic regression		
	Wald	*P*	OR (95%CI)	Wald	*P*	OR (95%CI)
Male	0.4814	0.4878	0.70 (0.25–1.93)			
Age (years)	0.3122	0.5763	0.99 (0.96–1.01)			
Disease duration (entero-BD)	0.3496	0.5543	0.99 (0.99–1.00)			
Multiple ulceration	1.5773	0.2091	1.96 (0.69–5.61)			
History of relapse	0.0121	0.9121	0.95 (0.36–2.48)			
History of perforation	4.0542	0.0345	0.23 (0.05–0.96)	2.4664	0.1163	0.28 (0.06–1.38)
History of surgery	1.8818	0.1701	0.35 (0.08–1.58)			
Concomitant CS use	0.5943	0.4407	1.46 (0.56–3.84)			
Concomitant CS dose (mg/day)	0.07655	0.7820	1.00 (0.98–1.03)			
Concomitant colchicine use	5.4007	0.0201	3.45 (1.21–9.80)	2.7596	0.0967	2.74 (0.73–8.99)
Concomitant drugs use (MTX, MS/SSZ, AZ)	3.4411	0.1007	3.44 (0.68–17.3)			
Concomitant MTX use	5.4008	0.0201	3.45 (1.21–9.80)	0.2392	0.6247	1.48 (0.30–7.23)
TNF-i use	7.6116	0.0017	6.60 (1.73–25.2))	3.8795	0.0416	6.17 (1.01–37.8)
DAIBD	0.5470	0.4595	1.01 (0.99–1.02)			

Model *χ*^2^ test *P* = 0.0040. *BD* Behcet’s disease, *CS* corticosteroid, *col* colchicine, *MS* mesalazine, *SSZ* sulfasalazine, *MTX* methotrexate, *AZ* azathioprine, *TNF-i* TNF inhibitors, *DAIBD* disease activity index for intestinal Behcet’s disease

### Effectiveness of TNF inhibitors on improvement of disease activity index for entero-Behcet’s disease

**Fig. 3:**
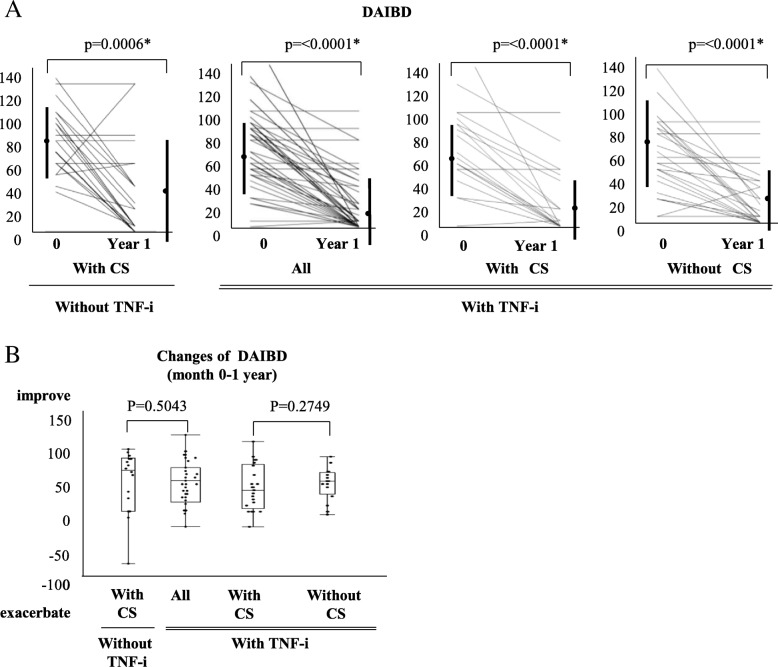
Effectiveness of TNF inhibitor in improvement of disease activity index for entero-Behcet’s disease. **a** DAIBD in the CS without TNF-i group (*n* = 22), TNF-i group (all: *n* = 49), TNF-i with CS group (*n* = 20), and TNF-i without CS group (*n* = 29). **b** Comparison of changes in DAIBD between the CS without TNF-i group (*n* = 22) and TNF-i group (all: *n* = 49), and between the TNF-i with CS group (*n* = 20) and TNF-i without CS group (*n* = 29) (by Wilcoxon signed-rank test). (last observation carried forward). Abbreviations: CS, corticosteroid; TNF-i, TNF inhibitors; DAIBD, disease activity index for intestinal Behcet’s disease

In the group with CS without TNF-i, the mean (± SD) DAIBD was 85.2 ± 29.6 at baseline and 40.5 ± 44.7 at 1 year, showing a significant decrease. In the group with TNF-i, the mean (± SD) DAIBD was also significantly decreased, from 64.7 ± 34.9 at baseline to 21.1 ± 28.9 at 1 year. In the group with TNF-i and CS, the mean (± SD) DAIBD was 70.7 ± 38.4 at introduction and 20.8 ± 29.3 at 1 year, demonstrating a significant decrease. Likewise, the mean (± SD) DAIBD was 60.5 ± 32.2 at introduction and 21.4 ± 25.5 at 1 year, showing a significant decrease in the group with TNF-i without CS (Fig. 3a). Comparison of the changes in DAIBD between the CS without TNF-i and TNF-i groups, and between the TNF-i with CS and TNF-i without CS groups, revealed no statistically significant differences (CS without TNF-i group 44.8 ± 47.1 vs. TNF-i 43.6 ± 37.2, TNF-i with CS group 50 ± 38.3 vs. TNF-i without CS group 39.1 ± 36.4) (Fig. 3b).

### Effectiveness of TNF inhibitor on reduction of concomitant CS dose

**Fig. 4:**
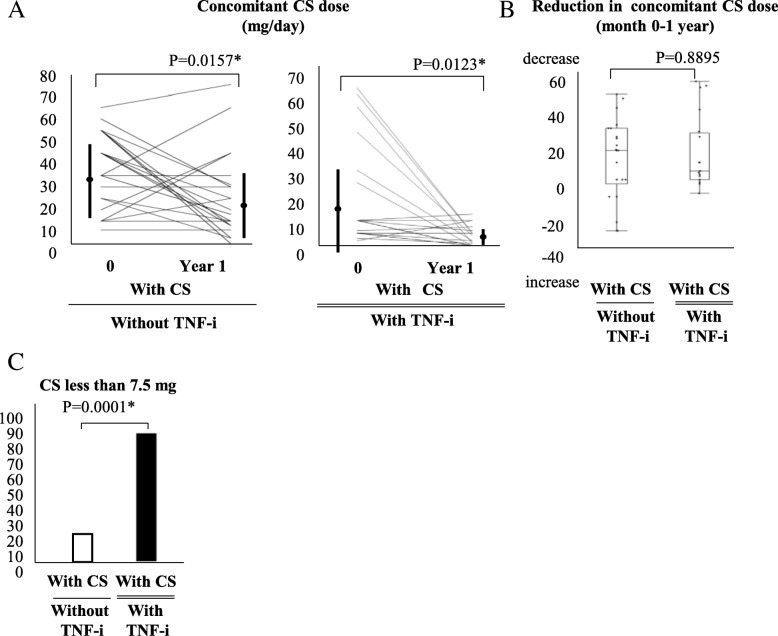
Effectiveness of TNF inhibitor in reduction of concomitant CS dose. **a** Concomitant CS dose in the CS without TNF-i group (*n* = 22) and TNF-i with CS group (*n* = 20). **b** Comparison of reduction of concomitant CS dose between the CS without TNF-i group (*n* = 22) and TNF-i with CS group (*n* = 20). (*P < 0.05, by Wilcoxon signed-rank test). **c** Comparison of proportion of concomitant CS dose < 7.5 mg. (*P < 0.05, by Fisher’s exact test) (last observation carried forward). Abbreviations: CS, corticosteroid; TNF-i, TNF inhibitors; DAIBD, disease activity index for intestinal Behcet’s disease

In the group with CS without TNF-i, the mean (± SD) dose of concomitant CS was 32.3 ± 16.4 mg/day at baseline and 20.6 ± 18.3 mg/day at 1 year, demonstrating a significant decrease. Likewise, the mean (± SD) dose of concomitant CS in the group with TNF-i and CS was 18.7 ± 20.9 mg/day at baseline and 3.88 ± 3.87 mg/day at 1 year, showing a significant decrease (Fig. 4a). Comparison of the reduction of concomitant CS dose between the CS without TNF-i group and the TNF-i with CS group demonstrated no statistically significant difference (CS without TNF-i group 11.7 ± 23.5 mg vs. TNF-i with CS group 14.9 ± 21.0 mg) (Fig. 4b). In contrast, the proportions of patients receiving concomitant CS dose less than 7.5 mg were significantly higher in the TNF-i with CS group than in the CS without TNF-i group (Fig. 4c). In the present study, achieving CS less than 7.5 mg/day at year 1 was observed in 21 patients. When baseline characteristics were compared between patients with achieving CS less than 7.5 mg/day or not, as shown in Table 4, MTX and TNF-i were significantly more frequently used and age was higher in patients with achieving CS less than 7.5 mg/day. In addition, univariate and multivariate logistic regression analyses were performed to identify the predictive factors achieving CS less than 7.5 mg/day (Table 5). As the results, although TNF-i is close to being significant, no evident predictive factor was found. Furthermore, there were not any statistical differences in the baseline factors and clinical effectiveness between patients with and without MTX in the TNF-i group (Tables 6 and 7).

**Table 4:** Comparison of baseline characteristics between the CS less than 7.5 mg/day group and not

Variables	CS ≦ 7.5 mg at year 1 (*n* = 21)	Not (*n* = 21)	*P value
Male, *n* (%)	6/21 (28.6%)	9/21 (42.9)	0.5204
Age (years)	42.8 ± 13.6	33.1 ± 10.5	0.0166*
Disease duration (entero-BD, months)	42.4 ± 68.8	40.3 ± 26.2	0.1341
Multiple ulceration, *n* (%)	4/21 (19.1%)	10/21 (47.6%)	0.1001
History of relapse, *n* (%)	11/21 (52.4%)	13/21 (61.9%)	0.7557
History of perforation, *n* (%)	3/21 (14.3%)	3/21 (14.3%)	1.0000
History of surgery, *n* (%)	4/21 (19.1%)	3/21 (14.3%)	1.0000
Concomitant CS dose (mg/day)	22.7 ± 21.5	28.9 ± 17.1	0.1450
Concomitant colchicine use, *n* (%)	15/21 (71.4%)	10/21 (47.6%)	0.2082
Concomitant drug use, *n* (%) (MTX, MS/SSZ, AZ)	18/21 (85.7%)	15/21 (71.4%)	0.4537
Concomitant MTX use, *n* (%)	15/21 (71.4%)	3/21 (14.3%)	0.0004*
TNF-i use, *n* (%)	17/21 (81.0%)	3/21 (14.3%)	< 0.0001*
DAIBD	70.0 ± 40.5	86.7 ± 25.3	0.0570

Plus-minus values are means ± standard deviation. *P < 0.05 by Fisher’s exact test or Mann–Whitney *U* test. *BD* Behcet’s disease, *CS* corticosteroid, *col* colchicine, *MS* mesalazine, *SSZ* sulfasalazine, *MTX* methotrexate, *AZ* azathioprine, *TNF-i* TNF inhibitors, *DAIBD* disease activity index for intestinal Behcet’s disease

**Table 5:** Baseline predictive factors of CS less than 7.5 mg/day analyzed by multivariate analysis

Variables	Univariate logistic regression			Multiple logistic regression		
	Wald	*P*	OR (95%CI)	Wald	*P*	OR (95%CI)
Male, *n* (%)	0.9232	0.3365	0.53 (0.15–1.92)			
Age (years)	5.1680	0.0230	1.07 (1.01–1.14)	2.32	0.1135	1.06 (0.98–1.13)
Disease duration (entero-BD, months)	0.0186	0.8914	1.00 (0.99–1.01)			
Multiple ulceration, *n* (%)	3.6556	0.1559	0.26 (0.06–1.03)			
History of relapse, *n* (%)	0.3876	0.5336	0.53 (0.43–5.05)			
History of perforation, *n* (%)	0	1.0000	1.00 (0.18–5.63)			
History of surgery, *n* (%)	0.1704	0.6797	0.71 (0.14–3.64)			
Concomitant CS dose (mg/day)	1.0678	0.3014	0.98 (0.95–1.02)			
Concomitant colchicine use, *n* (%)	2.4121	0.1139	2.75 (0.767–9.86)			
Concomitant drugs use, *n* (%) (MTX, MS/SSZ, AZ)	1.2318	0.2556	2.40 (0.51–11.3)			
Concomitant MTX use, *n* (%)	11.811	0.0006	15.0 (3.20–70.4)	2.32	0.5228	2.38 (0.16–34.2)
TNF-i use, *n* (%)	15.033	< 0.0001	25.5 (4.96–131.1)	3.11	0.0778	10.8 (0.77–153.0)
DAIBD	2.3752	0.1233	0.98 (0.97–1.00)			

Model *χ*^2^ test *P* < 0.0001. *BD* Behcet’s disease, *CS* corticosteroid, *col* colchicine, *MS* mesalazine, *SSZ* sulfasalazine, *MTX* methotrexate, *AZ* azathioprine, *TNF-i* TNF inhibitors, *DAIBD* disease activity index for intestinal Behcet’s disease

**Table 6:** Comparison of baseline characteristics between TNF-i with MTX (*n* = 40) and TNF-i without MTX (*n* = 9)

Variables	TNF-i without MTX (*n* = 9)	TNF-i with MTX (*n* = 40)	*P value
Male, *n* (%)	1 (11.1%)	11 (27.5%)	0.4203
Age (years)	43.7 ± 16.0	40.8 ± 13.7	0.2659
Disease duration (entero-BD, months)	54.0 ± 54.7	52.1 ± 63.5	0.5520
Multiple ulceration, *n* (%)	5/9 (55.6%)	27/40 (67.5%)	0.7001
History of relapse, *n* (%)	7/9 (77.8%)	22/40 (55.8%)	0.2771
History of perforation, *n* (%)	0/9 (0%)	9/40 (22.5%)	0.1795
History of surgery, *n* (%)	0/9 (0%)	6/40 (15.0%)	0.5765
Concomitant CS use, *n* (%)	6/9 (66.7%)	23/40 (57.5%)	0.7199
Concomitant CS dose (mg/day)	2.22 ± 3.63	8.83 ± 17.2	0.4414
DAIBD at baseline	61.1 ± 23.3	65.5 ± 37.3	0.8361

Plus-minus values are means ± standard deviation.*P < 0.05 by Fisher’s exact test or Mann–Whitney *U* test. *BD* Behcet’s disease, *CS* corticosteroid, *col* colchicine, *MS* mesalazine, *SSZ* sulfasalazine, *MTX* methotrexate, *AZ* azathioprine, *TNF-i* TNF inhibitors, *DAIBD* disease activity index for intestinal Behcet’s disease

**Table 7:** Comparison of clinical effectiveness between TNF-i with MTX (*n* = 40) and TNF-i without MTX (*n* = 9)

Variables	TNF-i without MTX (*n* = 9)	TNF-i with MTX (*n* = 40)	*P value
Cure of ulceration, *n* (%)	4/9 (44.4%)	21/40 (52.5%)	0.7252
Improvement of ulceration, *n* (%)	4/9 (44.4%)	23/40 (57.5%)	0.7126
Exacerbation of ulceration, *n* (%)	2/9 (22.2%)	10/40 (25%)	1.0000
Retention rate of TNF-i, *n* (%)	7/9 (77.8%)	33/40 (82.5%)	0.6631
Changes of DAIBD (month 0–1 year)	39.4 ± 35.7	44.5 ± 37.8	0.8460
Reduction in concomitant CS dose (month 0–1 year)	1.67 ± 3.53	6.13 ± 17.4	0.9191
CS less than 7.5 mg at year 1, *n* (%)	9/9 (100%)	36/40 (90.0%)	1.0000

Plus-minus values are means ± standard deviation. *P < 0.05 by Fisher’s exact test or Mann–Whitney *U* test. *BD* Behcet’s disease, *CS* corticosteroid, *col* colchicine, *MS* mesalazine, *SSZ* sulfasalazine, *MTX* methotrexate, *AZ* azathioprine, *TNF-i* TNF inhibitors, *DAIBD* disease activity index for intestinal Behcet’s disease

### Retention rate and adverse events of TNF inhibitor

The retention rates up to month 12 were 90.0% (18 of 20 patients) in the group with TNF-i and CS and 75.9% (22 of 29 patients) in the group with TNF-i without CS, demonstrating no significant differences (*P* = 0.1920). The status of 3 patients who newly started CS in the group with TNF-i without CS was classified as “Discontinuation.” The reasons for discontinuation are summarized in Fig. 1. During the observation period, mild infection (cold) occurred in 10 patients, cystitis in two, viral enteritis in two, influenza in one, tonsillitis in one, bronchitis in one, sinusitis in one, paronychia in one, and urticaria in one. One patient in the group with TNF-i and CS discontinued treatment owing to bacterial infection.

## Discussion

In the present study, we introduced TNF-i in 49 patients with refractory entero-BD and followed these patients for 1 year to assess the effectiveness of TNF-i. We also compared the effectiveness of TNF-i to that of CS without TNF-i. When the CS without TNF-i group and the TNF-i group were compared for effectiveness, the ulcer cure and improvement rates, as evaluated by lower gastrointestinal endoscopy, were also significantly lower in the CS without TNF-i group than in the TNF-i group (Fig. 2a, b). On the other hand, the DAIBD significantly decreased in both groups, with no statistically significant difference. As shown in Fig. 4c, the CS dose, which is considered a risk factor for gastrointestinal bleeding and perforation [16–18], was reduced to 7.5 mg or less in the majority of patients in the TNF-i group; however, in many patients in the CS without TNF-i group, dose reduction of concomitant CS was difficult despite a significant decrease in the DAIBD. In other words, patients in the CS without TNF-i group exhibited CS dependency.

At baseline, the CS without TNF-i group showed significantly higher DAIBD and CS dose than the TNF-i group. Regarding the differences at the baseline, we considered that past or recent treatment affected. Some patients had been treated with CS for other symptoms (e.g., skin, mucosa, eyes, genitourinary organs, joints) except entero-BD; some of the patients were under treatment with CS or under dose reduction of CS for entero-BD. For these patients, although we newly introduced TNF-i, choices regarding use and dose of CS were made at the physicians’ discretion. We considered that as one of the reasons of the difference at the baseline concomitant CS. On the other hand, although no evident predictive factor achieving CS less than 7.5 mg/day was found in our study (Tables 4 and 5), the multivariate analysis revealed that the TNF-i was an independent predictive factor for cure of the intestinal ulcerative lesions (Tables 2 and 3). Comparison of the groups clearly indicated that the use of TNF-i is important not only for reducing disease activity but also for achieving cure of ulcerative lesions.

The TNF-i group was further classified into two groups according to the presence or absence of concomitant CS use (TNF-i with CS group and TNF-i without CS group) to compare the effectiveness of TNF-i. The TNF-i formulations and the necessity of concomitant CS use were, in principle, determined at the discretion of the treating physicians. However, no significant differences in background factors were observed between the two groups at the time of TNF-i introduction (Table 1). Under these study conditions, no statistically significant differences between the TNF-i with CS group and the TNF-i without CS group were observed in any of the following endpoints: ulcer cure rate, ulcer improvement rate, ulcer exacerbation rate, retention rate of TNF-i, disease activity based on DAIBD, and decrease in DAIBD. Although none of the endpoints showed statistically significant differences, the respective rates or scores were also lower in the TNF-i without CS group than in the TNF-i with CS group. Furthermore, as shown in Fig. 1, the TNF-i with CS group included one non-responder to TNF-i, whereas the TNF-i without CS group included three. Although no patient in the TNF-i with CS group experienced relapse after introduction of TNF-i, four patients in the TNF-i without CS group did. No statistically significant differences were observed in the relapse rates or the proportions of non-responders (TNF-i with CS group 5.0%, TNF-i without CS group 13.8%, *P* = 0.3114, by Fisher’s exact test). Based on these results, although the activity of entero-BD was sufficiently controlled and maintained at low levels with TNF-i therapy without CS in some patients, there may be other patients for whom concomitant CS use is necessary. In addition, the present study failed to identify any background factors that could predict which patients would be non-responders or experience relapse in the TNF-i without CS group. Furthermore, we did not find any additive effect of MTX on TNF-i (Tables 6 and 7). In the future, additional studies with a larger sample size may be needed to reveal the features of patients who do or do not require a combination of TNF-i and CS. As shown in Fig. 3, concomitant CS doses were significantly reduced in the TNF-i with CS group. Moreover, as described above, the doses were reduced to 7.5 mg or lower in most patients in the TNF-i with CS group, unlike in the CS without TNF-i CS group. During the observation period, neither serious gastrointestinal bleeding nor perforation occurred in any patients in the TNF-i group. In contrast, one patient in the TNF-i with CS group developed bacterial infection resulting in discontinuation of treatment (this patient had entero-BD associated with myelodysplastic syndrome involving trisomy 8). In contrast, no patients in the TNF-i without CS group developed infection requiring discontinuation of TNF-i. Thus, although combination therapy with CS and TNF-i necessitates caution to prevent infection, the current study suggested that TNF-i therapy without CS might be beneficial in reducing the risk of infection.

Limitations of the present study include a small sample size (49 patients) and a short retrospective observation period (1 year). In the future, studies with a larger sample size and a longer observation period are warranted to assess the long-term efficacy of TNF-i in controlling disease activity and to identify predictive factors to detect patients who do not require concomitant usage of CS.

## Conclusions

In the present study, we administered TNF-i to 49 patients with refractory entero-BD and demonstrated its high efficacy based on both endoscopy results and a quantitative disease activity index. In particular, comparison between the TNF-i group and the CS without TNF-i group revealed that TNF-i is highly effective in curing ulcers and reducing concomitant CS doses and identified as an independent predictive factor for cure of the intestinal ulcerative lesions. Furthermore, TNF-i demonstrated sufficient efficacy in both the TNF-i with CS group and the TNF-i without CS group to control disease activity without concomitant CS in some patients. In contrast, TNF-i therapy with CS was associated with concerns regarding infection, and TNF-i therapy without CS with concerns regarding non-responders and relapse.

## Data Availability

Not applicable.

## Abbreviations

BD: Behcet’s disease
CS: Corticosteroid
DAIBD: Disease activity index for intestinal Behcet’s disease
entero-BD: Entero-Behcet’s disease
HLA: Human leukocyte antigen
TNF: Tumor necrosis factor
TNF-i: TNF inhibitor
